# Estimated Cost Effectiveness of Influenza Vaccination for Emergency Medical Services Professionals

**DOI:** 10.5811/westjem.2021.7.50681

**Published:** 2021-10-26

**Authors:** Michael W. Hubble, Ginny K. Renkiewicz

**Affiliations:** Wake Technical Community College, Department of Emergency Medical Science, Raleigh, North Carolina

## Abstract

**Introduction:**

Because of their frequent contact with compromised patients, vaccination against influenza is recommended for all healthcare workers. Recent studies suggest that vaccination decreases influenza transmission to patients and reduces worker illness and absenteeism. However, few emergency medical services (EMS) agencies provide annual vaccination, and the vaccination rate among EMS personnel remains low. Reticence among EMS agencies to provide influenza vaccination to their employees may be due in part to the unknown fiscal consequences of implementing a vaccination program. In this study, we sought to estimate the cost effectiveness of an employer-provided influenza vaccination program for EMS personnel.

**Methods:**

Using data from published reports on influenza vaccination, we developed a cost-effectiveness model of vaccination for a hypothesized EMS system of 100 employees. Model inputs included vaccination costs, vaccination rate, infection rate, costs associated with absenteeism, lost productivity due to working while ill (presenteeism), and medical care for treating illness. To assess the robustness of the model we performed a series of sensitivity analyses on the input variables.

**Results:**

The proportion of employees contracting influenza or influenza-like illness (ILI) was estimated at 19% among vaccinated employees compared to 26% among non-vaccinated employees. The costs of the vaccine, consumables, and employee time for vaccination totaled $44.19 per vaccinated employee, with a total system cost of $4,419. Compared to no vaccination, a mandatory vaccination program would save $20,745 in lost productivity and medical costs, or $16,325 in net savings after accounting for vaccination costs. The savings were 3.7 times the cost of the vaccination program and were derived from avoided absenteeism ($7,988), avoided presenteeism productivity losses ($10,303), and avoided medical costs of treating employees with influenza/ILI ($2,454). Through sensitivity analyses the model was verified to be robust across a wide range of input variable assumptions. The net monetary benefits were positive across all ranges of input assumptions, but cost savings were most sensitive to the vaccination uptake rate, ILI rate, and presenteeism productivity losses.

**Conclusion:**

This cost-effectiveness analysis suggests that an employer-provided influenza vaccination program is a financially favorable strategy for reducing costs associated with influenza/ILI employee absenteeism, presenteeism, and medical care.

## INTRODUCTION

Influenza is a significant disease in the United States that contributed to approximately 44.8 million illnesses, 808,129 hospitalizations, and 61,099 deaths during the 2017–2018 influenza season.^1^ Annual immunization is recommended for all persons over six months of age^2^ and is the best prevention against contracting influenza or experiencing severe illness if infected. Moreover, vaccination of healthcare workers (HCW) has been shown to decrease influenza transmission to patients, as well as reduce worker illness.^3^ For these reasons, the Advisory Committee on Immunization Practices and the Healthcare Infection Control Practices Advisory Committee recommend that all US healthcare workers be vaccinated annually against influenza.^2^,^4^

There are approximately 248,000 emergency medical services (EMS) personnel in the US who are on the front lines of patient care and may play a significant role in the transmission of influenza to patients and co-workers.^5^,^6^ One estimate reports that during an influenza season, as many as 12% of all patients with influenza-like illness (ILI) treated in an emergency department arrived via EMS,^7^ which indicates a significant exposure risk for EMS personnel. Once infected, an employee can transmit the disease one day prior to the onset of symptoms,^8^ and as many as 40% of healthcare workers purposefully continue to work while they are ill—a phenomenon known as presenteeism.^9^,^10^,^11^ During presenteeism, clinicians may see an overall decrease in productivity, increased medical errors, and impaired clinical judgment.^11^ Additionally, EMS employees may unwittingly transmit influenza to high-risk patients as well as coworkers and members of their own families.

There is scant literature regarding barriers to vaccination in EMS agencies, although vaccination cost and lack of availability in the workplace have been cited.^6^ While employer promotional efforts appear to have a direct correlation with vaccination rates, vaccination coverage remains lower than ideal in this population.^7^,^12^,^13^ EMS professionals are 27 times more likely to obtain the influenza vaccine when they believe the vaccine is safe and over three times more likely when a vaccination program is available through their employer.^12^ Unlike hospitals, where mandatory immunization programs are becoming more commonplace, such programs among EMS agencies are relatively uncommon.^12^ Little is known about the rationale that underlies the lack of mandated vaccination programs in EMS agencies, although one possibility is that the cost effectiveness of such programs is largely unknown.

Reports of the cost effectiveness of influenza vaccination in EMS are lacking despite the presence of similar studies conducted among other healthcare settings. These evaluations of cost effectiveness were conducted from the employer’s perspective and focused on the prevention of absenteeism and medical care costs for treating illness as the primary benefits of immunization. Nonetheless, there was variability in methodology and worker population, including differences in the cost parameters used across the studies. To further inform EMS administrators who must develop programs or policies regarding influenza immunization, we sought to develop a deterministic cost-effectiveness model of a mandatory, employer-provided immunization program from the financial perspective of the EMS agency.

## METHODS

This project received institutional review board approval from Wake Technical Community College, Department of Emergency Medical Science. Using estimates from the published literature on influenza vaccination and illness, we developed a deterministic cost-effectiveness model of an employer-provided vaccination program from the perspective of the EMS employer. We chose a deterministic model rather than a probabilistic or simulation model because the former can easily be replicated with local data by an EMS manager using only a spreadsheet, whereas the latter modeling techniques require knowledge of statistical methods and computer programming languages. We calculated the cost to vaccinate an individual and then extrapolated the cost to a hypothesized EMS system of 100 employees. Model inputs included vaccination costs, vaccine uptake rate, infection rate, and costs associated with absenteeism, lost productivity due to working while ill (presenteeism), and medical care for treating illness (e.g., medical office visits and prescription drugs). To assess the robustness of the model we performed a series of sensitivity analyses on the input variables.

**Population Health Research Capsule**

What do we already know about this issue?

*Vaccination decreases influenza transmission to patients and reduces worker illness and absenteeism.*

What was the research question?

*What is the cost effectiveness of an employer-provided influenza vaccination program for emergency medical service (EMS) personnel?*

What was the major finding of the study?

*Employer-provided influenza vaccination is a cost-effective means for reducing absenteeism, presenteeism, and medical care.*

How does this improve population health?

*Investments in an employer-provided influenza vaccination program can reduce absenteeism and promote response readiness of the implementing EMS agency.*

### Estimation of Vaccination Costs

The costs of implementing influenza vaccination include the vaccine itself and disposable supplies (e.g., needles, syringes, and gloves), which was modeled at $21.42 per employee.^14^ In addition, we assumed 15 minutes of time for each vaccine administration by an infection control nurse,^15^ as well as 20 minutes of lost work time for the vaccine recipient.^16^ Personnel costs were calculated using mean hourly wages plus 30% benefit costs for registered nurses and paramedics.^17^,^18^ Paramedic compensation was calculated at $26.53 per hour, totaling $8.84 per vaccination, and registered nurse compensation was calculated at $55.71 per hour, totaling $13.93 per vaccination. Costs for each vaccination including vaccine, supplies, and employee compensation totaled $44.19 for each vaccinated employee.

### Estimation of Vaccine Uptake Rate

The vaccine uptake rate describes the willingness of a target population to engage or participate in vaccination programs and is not extensively documented among EMS personnel. Among the few published reports, rates varied from a low of 21% as reported by Rueckmann et al.^6^ to a high of 100% as reported by Rebmann et al.^3^ Of particular interest is that the uptake rate of 100% was obtained as the result of an employer-mandated vaccination program. When not employer-mandated, vaccination participation rates ranged from 21% to 66.8% for EMS personnel.^3^,^6^,^12^ For our model, we assumed a mandatory vaccination policy for which the EMS employer would provide vaccinations for all employees and would bear all associated costs.

### Estimation of Vaccine Effectiveness

We calculated an estimation of vaccine effectiveness as a weighted average of published case series across several influenza seasons and varying degrees of match between vaccine and circulating strains. To more accurately capture the exposure risk we limited the studies used in the calculation of vaccine effectiveness to those among healthcare workers rather than the general working adult population.^19^–^26^ From these studies, we modeled the ILI rate of vaccinated employees at 18.97% and 25.74% for unvaccinated employees.

### Estimation of Illness Costs

The costs of influenza and ILI in the workplace are derived from several different factors, which include absenteeism, presenteeism, and necessary medical care for treating the illness. Our model for employer costs is based on the mean salary for the paramedic, including an additional 30% for benefits,^18^ and we assumed all work shifts to be 12 hours in length. We did not explicitly account for any additional costs associated with backfilling absentee shifts with full-time or part-time personnel, although these costs are acknowledged.

Based upon published reports, we modeled the weighted-average days of lost work time for unvaccinated vs vaccinated healthcare and other workers at 2.87 and 2.57 days, respectively.^19^,^21^,^22^,^27^–^29^

Healthcare workers are more inclined to report to work while ill compared to other professional groups,^10^,^11^,^30^ and presenteeism is estimated to cost $2,000–$15,541 annually per healthcare employee.^31^ Among all workforce sectors, the cost of presenteeism to employers in the US is nearly $150 billion dollars per year.^32^ We incorporated this productivity loss in our model using published estimates of the mean days of presenteeism for vaccinated (3.93 days) and unvaccinated (5.63 days) healthcare workers,^29^ with employee productivity during presenteeism shifts estimated at 54% of normal.^33^

Using a weighted average from published reports, we estimated that 35.8% of vaccinated employees with ILI would seek medical treatment; however, that rate was 52.73% in unvaccinated employees.^21^,^27^,^34^ Medical costs for the treatment of influenza and ILI were estimated at $362 per person, not including the cost of over-the-counter medications. This estimate was based on actual costs reported by Soni and Hill,^35^ which were converted to 2019 dollars using the Medical Cost Inflator.^36^

### Sensitivity Analysis

To assess the robustness of our model we performed a series of univariate and bivariate sensitivity analyses by modifying input variables to assess the impact on cost effectiveness. These variables of interest were vaccination costs, employee infection rate, presenteeism, and absenteeism. The relevant ranges across which these variables were established used speculative ranges of 0–100% for variables of proportions (vaccine uptake rate and lost productivity of presenteeism) and ranges of ± 10% of the point estimate for all other variables. In our univariate sensitivity analyses, vaccination costs varied between $35.45 and $48.61, the vaccination uptake rate was varied from 0–100%, and the presenteeism lost productivity rate was varied between 0–100%.

We performed two-way sensitivity analyses on the ILI rate, missed days of work, and presenteeism days. The rate of employees suffering from influenza or ILI was simultaneously varied between 23.17–28.31% for unvaccinated workers and between 17.07–20.87% for vaccinated workers. Lost workdays were simultaneously varied between 2.58 and 3.16, and between 2.31 and 2.83 days for unvaccinated and vaccinated workers, respectively. Similarly, presenteeism shifts were simultaneously varied between 5.07 and 6.19 shifts, and 3.54 and 4.32 shifts for unvaccinated and vaccinated workers, respectively.

## RESULTS

### Base-Case Scenario

For the base-case scenario, we assumed that an influenza vaccination program was neither in place nor offered to the employees and that no employees had obtained vaccination of their own volition. We anticipated that all ILI-related treatment costs were ultimately borne directly by the employer. In our hypothesized agency of 100 unvaccinated employees, 26 were expected to be affected by influenza or ILI, which caused 2.86 missed shifts per employee, resulting in a total of 73.78 lost shifts for the agency overall. This absenteeism represents $23,490 in lost productivity, assuming that employees were compensated via sick-day benefits. Regarding the impact of presenteeism in this hypothesized population, reduced productivity persisted over 5.63 shifts per employee, resulting in a total of 146.38 shifts overall for the agency. Productivity during a presenteeism shift was estimated at just 54% of normal,^33^ resulting in a cost to the employer of $21,221. Ill employees would have also amassed $4,912 in associated healthcare costs. The total cost of influenza for the hypothetical agency was estimated at $49,623 annually (Table 1).

### Mandatory Vaccination Scenario

Once a baseline was established, we repeated the scenario with the assumption that the hypothesized agency had a 100% vaccination uptake rate through an employer-mandated vaccination program. In this scenario, absenteeism affected just 19 employees, which caused 2.57 missed shifts per vaccinated employee, and resulted in 48.69 missed shifts for the agency overall. In comparison to the unvaccinated workforce, this represents a reduction in the cost of lost workdays of $7,988. Presenteeism also declined for the vaccinated group with just 3.93 shifts per vaccinated employee, for a total of 74.67 shifts for the agency overall. Assuming the same degree of reduced productivity during a period of presenteeism (54% of normal), this intermediate stage of productivity would have a total cost of $10,918, yielding an annual savings of $10,303. Additionally, this scenario also produced a decrease in ILI-associated healthcare costs of $2,454 ($2,458 vs $4,912). Overall, the annual net savings from a mandatory vaccination program was $16,325, which is approximately 3.7 times the cost of the overall program.

### Sensitivity Analysis

We performed univariate sensitivity analyses on the vaccination uptake rate, vaccination cost, and presenteeism productivity-loss variables. The net savings to the employer were sensitive to the vaccination uptake rate, which is unlikely to be 100% even under a mandatory vaccination program. Additionally, some employees may receive vaccinations outside of their employer-sponsored program. Consequently, when the uptake rate was varied between 0–100%, the net savings ranged from $0–$16,326 over the base-case scenario.

Variation of the costs per vaccination from $35.35 to $48.61 (± 10% of the base case) resulted in net savings between $15,884 and $17,210, which indicated that the economic benefits of vaccination are comparatively insensitive to this cost driver. The presenteeism productivity loss had a substantial impact on net savings. As this variable was adjusted between 0–100%, the net savings ranged between $3,509 and $27,244.

We performed two-way sensitivity analyses on the ILI rate and the number of absenteeism and presenteeism shifts per employee. For the sensitivity analysis of the proportion of employees anticipated to acquire ILI, the proportion for unvaccinated and vaccinated employees were varied simultaneously by ± 10% of the base case. The net savings were $8,481 for the worst-case scenario (23.17% and 20.87% ILI rates for unvaccinated and vaccinated employees, respectively), and $24,182 for the best-case scenario (28.31% and 17.07%, respectively), suggesting that the net savings are sensitive to the ILI rate difference between vaccinated and unvaccinated workers.

The prevailing literature suggests that there is little difference between vaccinated and unvaccinated workers in the number of work shifts missed once they develop ILI. Predominate factors for absenteeism are low pay and available time off, whereas working ill is associated with endorsement of presenteeism in the workplace culture, reluctance to burden coworkers, and associating being at work with competence.^11^ Consequently, the net cost savings were only marginally sensitive to absenteeism. As this variable was simultaneously adjusted by ± 10% of the base case, the net savings spanned from $12,429 to $20,237 between the most favorable and unfavorable scenarios.

Although the literature suggests that the difference in the number of presenteeism shifts for each ill employee are more striking than the number of absenteeism shifts between vaccinated and unvaccinated workers, the monetary consequence is somewhat moderated by the fact that the employees remain productive, albeit at reduced levels. As the number of presenteeism shifts were simultaneously modified by ± 10% of the base case, the net savings spanned from $13,112 to $19,540 between the most favorable and unfavorable scenarios. Sensitivity analysis results are summarized in Table 2.

## DISCUSSION

When not employer-mandated, vaccination participation rates among EMS professionals remain low,^3^,^6^,^12^ although vaccination is a proven means of disease prevention.^37^,^38^ Low immunization coverage among EMS professionals poses a risk to hospitalized and long-term care patients who are already vulnerable to nosocomial infection. In addition to the societal costs of influenza and ILI in terms of morbidity and mortality, illness among the EMS workforce creates an economic burden for the employer via absenteeism, presenteeism, and medical care costs, some of which may be mitigated by a mandatory, employer-sponsored vaccination program. Although vaccine hesitancy continues to be an issue among some healthcare workers, most hospitals mandate influenza vaccination—a policy change that has resulted in immunization coverage rates in excess of 90% for clinicians.^39^–^41^ However, mandatory influenza vaccination is rare among EMS agencies despite the significant risk of disease transfer to vulnerable populations.^12^ One possible factor contributing to the lack of mandatory vaccination programs in EMS is the lack of proven cost-effectiveness for the EMS agencies employing these professionals.

In the absence of a controlled influenza vaccination trial designed to demonstrate the cost effectiveness of reducing EMS employee illness and the consequences of that illness, the potential benefits can only be estimated indirectly using historical data. Based on published estimates of vaccination costs, ILI rates, treatment costs, and lost productivity among ill workers, our model suggests that the mandatory vaccination of EMS professionals is a cost-effective strategy for reducing financial losses associated with influenza and ILI in the EMS workforce. For a hypothetical EMS system of 100 employees, the total cost of vaccination including the vaccine, supplies, and employee compensation would equal $4,419 or $44.19 per vaccinated employee. In return, the net savings from reduced absenteeism, presenteeism, and avoided medical costs was $16,325—or $163 per vaccinated employee, a total that is nearly four times the cost of the overall program.

Although our model was based on a hypothetical EMS system of 100 employees, the model was structured such that the input and output variables are linear and scalable. Consequently, the crude cost-effectiveness of universal vaccination can be easily estimated for an EMS system of any size by using the per vaccinated employee cost ($44) and net savings ($163) point estimates. While actual realized savings may vary, our estimates were verified across a series of sensitivity analyses and should serve as a reasonable approximation. Notably, even under the most pessimistic assumptions, there were still cost savings for the employer.

Although we were unable to identify any previous reports of cost-effectiveness studies of influenza vaccination among EMS agencies, similar studies have been conducted among other healthcare settings. Ito et al. found that the cost of vaccination was lower than the cost of one day of absenteeism; however, only disposable supplies and the employee’s and nurse’s time for immunization were included, and the study did not account for the cost of the vaccine itself.^22^ In one of the more comprehensive analyses, Meijboom et al. included the cost of vaccine, employee and nurse time for vaccination, supplies, overhead for implementing the vaccination program, productivity losses, and medical costs resulting from adverse events of vaccination, as well as medical costs for treating in-hospital patients with hospital-acquired infection via an infected HCW.^42^ They found the program to be cost effective despite assuming an HCW vaccine coverage rate of only 15.47%.

In a literature review of worksite influenza immunization programs, Olsen et al. reviewed two randomized trials and four cost-benefit models based on non-HCWs.^43^ The authors concluded that such programs were generally cost effective, with the primary savings derived from avoided lost productivity rather than averted healthcare costs for those with influenza in the workplace.

In an analysis more similar to ours in terms of methodology, Colombo et al. evaluated the cost effective-ness of an influenza vaccination program at an Italian public healthcare unit.^19^ As cost inputs, this study included the cost of vaccine, supplies, nurse and physician time for administration, and employee time for vaccine receipt. Vaccination program benefits included cost savings from reduced absenteeism but not from reduced presenteeism or avoided treatment costs of sick employees. A cost-benefit ratio of 4.2 was reported, which was similar to our ratio of 3.7 despite some differences in model assumptions.

Although our results suggest that vaccination is cost effective, a mandatory vaccination program for EMS professionals holds potential for reducing nosocomial infection among EMS patients as well as other patients encountered by EMS in the hospitals and long-term care facilities they frequent. This secondary benefit may be of greater importance than the potential direct cost savings from avoided workforce illness, and vaccination of EMS professionals could be justified on this basis alone even if the vaccination program resulted in a net cost. Prior studies have demonstrated that up to 25% of HCWs are infected with influenza during the season of prime prevalence and those who are ill seldom stay away from work.^26^,^44^ Additionally, some infected employees are asymptomatic, yet shed influenza virus. Consequently, the working ill and subclinically infected workers can perpetuate influenza transmission within healthcare facilities. This is particularly true of EMS professionals given the tighter working quarters and the known transmission of influenza from respiratory particulates that can occur within a six-foot radius.^8^ Thus, a mandatory vaccination program for EMS professionals may convey monetary rewards that extend well beyond those directly benefiting the EMS employer.

## LIMITATIONS

The purpose of this study was to estimate the cost effectiveness of a mandatory vaccination program while accounting for costs borne solely by the employer. This model does not attempt to address the costs to society. More importantly, this model did not account for the financial and human suffering costs associated with the unintended spread of influenza from EMS caregivers to others. The model did not attempt to quantify the value to vaccinated workers who contract influenza but have a milder manifestation of disease—a limitation that may underemphasize the efficacy and merit of such programs. It also did not incorporate the indirect benefits of vaccination linked to herd immunity.

Direct evaluation of the benefits of vaccination programs among EMS workers are lacking. Consequently, our calculations are largely theoretical and based, in part, upon previously published data. Future research should seek to address the explicit costs of vaccination programs implemented in EMS agencies.

The accuracy of our cost-effectiveness estimates was limited by the precision of our input variables drawn from the literature. The analysis used infection rates of vaccinated workers over multiple years, varying from 1.6% up to nearly 63%, with the mean infection rate being 18.9%. As a result, our estimates are what should be expected for an “average” vaccine match but cannot account for other confounding variables, such as particularly virulent strains, individual susceptibility to infection, or other environmental factors that confer a higher predisposition for contraction of influenza. Consequently, our effectiveness estimates are generalizable to annual vaccination programs during periods of typical antigenic drift, but we caution against extrapolating these results to any season with a pandemic strain.

We did not account for productivity losses or treatment costs associated with adverse effects related to vaccination. However, most adverse events are of minimal medical consequence and serious sequelae are rare. Thus, adverse events would be unlikely to substantially alter our conclusions.^45^–^47^ Additionally, because the purpose of this study was to provide a cost-effectiveness analysis for the employer, we did not assess the effects of such a program on minimizing transmission, morbidity, or mortality of the disease. Finally, we did not account for such measures as Quality-Adjusted Life Years or Disability-Adjusted Life Years that may be pertinent to illness contracted by EMS personnel.

## CONCLUSIONS

This cost-effectiveness analysis suggests that an employer-provided influenza vaccination program is a cost-effective strategy for EMS agencies. Based upon our hypothetical model of an EMS system with 100 employees, the implementation of a mandatory vaccination program may produce savings of up to 34% in lost wages, 49% in reduced productivity, and a 50% reduction in associated healthcare costs. This model may be useful for EMS agency managers investigating the feasibility and cost effectiveness of implementing such a program, particularly in light of the COVID-19 pandemic. Additional research should focus on the direct measurement of cost effectiveness of vaccination as well as the attitudes and beliefs of EMS professionals related to vaccination for influenza and COVID-19 to create a holistic understanding of vaccination programs within the EMS workforce.

## Figures and Tables

**Table 1:** Cost-effectiveness analysis regarding employer-paid influenza vaccinations for paramedics.

Input variables	Scenario	

	Base case no vaccination	Universal vaccination
Personnel variables		
Total number of personnel	100	100
Vaccine uptake rate	0%	100%
Length of shift	12	12
Paramedic hourly pay rate	$26.53	$26.53
Vaccination variables		
Cost of vaccine	$0	$18.42
Cost of supplies	$0	$3.00
Cost of vaccine administration		
Infection control nurse (15 minutes at $55.71/hour)	$0	$13.93
Paramedic employee (20 minutes at $26.53/hour)	$0	$8.84
Vaccination costs per employee	$0	$44.19
Total vaccination cost	$0.00	$4,419.15
Vaccine effectiveness		
Proportion of employees with influenza-like illness	25.74%	18.97%
Number of employees with influenza-like illness		
Vaccinated	0	19
Unvaccinated	26	0
Costs due to lost productivity		
Lost productivity due to absenteeism		
Number of shifts missed due to illness per ill employee	2.87	2.57
Total number of shifts missed due to influenza-like illness	73.78	48.69
Cost of missed shifts due to influenza-like illness	$23,490	$15,501
Lost productivity due to presenteeism		
Number of days of presenteeism per employee	5.63	3.93
Total number of days of presenteeism	145	75
Total number of shift hours of presenteeism	1739	895
Lost productivity rate due to presenteeism	46%	46%
Total hours of productivity lost to presenteeism	800	412
Total cost of lost productivity due to presenteeism	$21,221	$10,918
Health care costs of treating influenza-like illness		
Proportion of employees seeking medical care	52.73%	35.80%
Number of employees seeking medical care	14	7
Medical treatment costs per employee	$362	$362
Total medical care costs	$4,912	$2,458
Cost effectiveness		
Total costs of vaccination	$0	$4,419
Total costs of absenteeism, presenteeism, and medical care	$49,623	$28,878
Total employer costs	$49,623	$33,297
Net savings from vaccination		$16,325

**Table 2:** Results of sensitivity analysis.

Variable (base case)	Range varied	Savings for the employer
Vaccination uptake rate (0%)	0% – 100%	$0–$16,325
Vaccination costs ($44.19)	$35.35 – $48.61	$17,210–$15,884
Proportion of employees with influenza-like Illness		
Unvaccinated (26%)	23.17% – 28.31%	$8,481 – $24,182
Vaccinated (19%)	17.07% – 20.87%	
Absenteeism shifts per ill employee		
Unvaccinated (2.87)	2.58–3.16	$12,429–$20,237
Vaccinated (2.57)	2.31–2.83	
Presenteeism shifts per ill employee		
Unvaccinated (5.63)	5.07–6.19	$13,112–$19,540
Vaccinated (3.93)	3.54–4.32	
Presenteeism productivity loss (46%)	0% – 100%	$3,509–$27,244
